# Community-involved economic evaluation and development of a cost-effectiveness calculator for two peer-driven substance use interventions

**DOI:** 10.3389/fpubh.2025.1519980

**Published:** 2025-09-11

**Authors:** Sierra Castedo de Martell, Margaret B. Moore, Hannah Wang, Lori Holleran Steiker, J. Michael Wilkerson, Sheryl A. McCurdy, Nalini Ranjit, H. Shelton Brown

**Affiliations:** ^1^Research Department, The Phoenix, Denver, CO, United States; ^2^School of Public Health, The University of Texas Health Science Center at Houston, Houston, TX, United States; ^3^Steve Hicks School of Social Work, The University of Texas at Austin, Austin, TX, United States

**Keywords:** cost-effectiveness analysis, community-based participatory research, community-involved research, economic evaluation, peer recovery support services

## Abstract

**Introduction:**

While peer-driven substance use interventions have proliferated across the U.S., economic evaluations of these interventions have lagged behind. A key characteristic of these interventions is the centrality of the “nothing about us without us” ethos, which should extend into economic evaluation research. To that end, this study sought to take a community-based participatory research (CBPR) approach to conducting a cost-effectiveness analysis (CEA) of peer recovery support services (PRSS) and turning that CEA and a CEA of bystander naloxone distribution (BND) into components of a free, web-based calculator for use by recovery community centers (RCCs).

**Methods:**

We engaged staff and administrators (*n* = 10) at two RCCs as community partners. We developed preliminary analytic models for the CEAs and engaged the RCCs in a feedback session to inform the final CEA models. We then built prototype calculators and pre-tested them with our community partners. After integrating all feedback, we launched the pilot calculator for PRSS and BND CEA and have continued to collect feedback.

**Results:**

Our RCC community partners substantively and meaningfully engaged in the co-creation of the CEA calculator and the analytic model. Calculator users have largely rated the calculator somewhat to very easy to use (58.33% and 29.17%, respectively), and rated the interpretability of results as neutral (25%), somewhat easy (45.83%) to very easy (20.83%), while finding the required information to input into the calculator was more challenging, with 8.33% rating it very difficult, 4.17% somewhat difficult, 37.5% neutral, 41.67% somewhat easy, and only 8.33% rating it very easy. There was broad agreement that calculator results would be useful for their organizations (20.83% neutral, 41.67% somewhat useful, 37.5% very useful).

**Discussion:**

RCCs face known challenges with data collection and management. This study was limited by its size (10 live participants and 24 post-launch feedback surveys). However, feedback is continuing to be collected, and a larger-scale future study is planned.

**Conclusion:**

This project demonstrates that it is feasible to take a CBPR approach to economic evaluation, and that both scholarly research and easily-interpretable tools can be created from such an approach that mutually benefits researchers and community organizations.

## 1 Introduction

Despite the recent proliferation of peer-driven interventions to reduce the harms of substance use and support recovery, economic evaluations of these peer-driven interventions have lagged behind (1, 2). A majority of the economic evaluation literature on substance use interventions focuses on pharmaceutical or clinical interventions (3–16), rather than those driven by peers, though there are exceptions: a recent cost-effectiveness analysis (CEA) of long-term peer recovery support services (PRSS) and a CEA of bystander naloxone distribution (BND) from 2013 (17, 18). Economic evaluation information like CEA is important to inform policy decisions by guiding funders on how to allocate limited resources to address health problems, but ultimately it is individuals affected by these health problems who bear the brunt of these decisions (19). That is why a recent update to the Consolidated Health Economic Evaluation Reporting Standards (CHEERS) statement included guidance on including the voices of patients and the public in economic evaluations as a standard practice (19). This push is particularly salient to the closure of the economic evaluation gap surrounding peer-driven interventions for substance use, as many of these interventions operate with a core principle of “nothing about us without us,” which must necessarily extend into economic evaluations of these programs.

A substantial challenge to the accessibility of economic evaluation information by patients and the public is the interpretability of evaluation results. Often, such results appear only in the academic literature, where—if not behind a paywall—Staniszewska and colleagues (19) note “the language of health economic evaluation is complex and not always accessible, creating challenges for meaningful public involvement in key deliberation and discussion.” Readers who are not health economists may be challenged by how to interpret the generalizability of findings: for example, if the analysis only involved sites in large cities, then would the same results be true in smaller towns or in rural areas? While sensitivity analyses can help address questions around scale and around variability in on-the-ground conditions, providing tailored economic evaluation results based on specific, real-world inputs could help bridge the interpretability gap, and increase the accessibility of economic evaluation information after an analysis has been completed.

To address these twin challenges in economic evaluations of peer-driven substance use interventions, we took a community-based participatory research (CBPR) approach to building a web-based pilot *cost-effectiveness calculator* that provides tailored, base case CEA results based on user inputs with plain language results interpretation. The pilot calculator includes two components: one component based off the 2013 BND CEA (18), and one based on a CEA of PRSS performed by the research team with community partners as part of this project (17). Both components were selected because they are commonly-employed service offerings at recovery community centers (RCCs)—key hubs of peer-driven substance use interventions in communities across the U.S. (20). This project was supported by a grant through the Recovery Research Institute’s pilot grant program (via NIDA R24DA051988) supporting research on RCCs, and builds upon previous work developing a calculator tool for collegiate recovery program cost-effectiveness (21).

## 2 Materials and methods

This study was reviewed, declared exempt, and approved by the Committee for the Protection of Human Subjects at the University of Texas Health Science Center at Houston (IRB approval number HSC-SPH-21-1057). The study was declared exempt under category 2 (typical survey procedures not involving personally identifiable information) and category 4 (only secondary, de-identified or publicly-available data used to conduct PRSS CEA). The PRSS CEA was further reviewed, declared as having no human subjects, and approved by the same committee (IRB approval number HSC-SPH-21-0768).

### 2.1 Overview and team roles

The research team consisted of coauthors SCM, HSB, MBM, and HW, in collaboration with a total of 10 staff and administrators at two RCCs in Austin, Texas: Communities for Recovery, and RecoveryATX. Both centers offered PRSS and BND. RCCs were not monetarily compensated for their time, but instead were compensated in-kind by receiving customized results interpretation for the cost-effectiveness of their PRSS and BND programs for use in reports and proposals at the end of the study period. SCM led the PRSS component, MBM led the BND component, HW was responsible for conversion of all calculator prototypes (Excel-based) to a web-based format (HTML-based) and testing for compliance with the Americans with Disabilities Act (ADA). HSB oversaw the project and developed the quality-adjusted life expectancy estimator for both calculator prototype components. SCM is also a person in recovery from SUD, and delivered PRSS prior to moving into research.

### 2.2 Analytic model development

Because we developed the PRSS component *de novo*, we first performed a systematic review to identify potential parameters in the literature, and to create a preliminary analytic model prior to meeting with community partners. The systematic review search phase was completed in 2022 and was published in 2024 (22). A full parameter list and detailed description of the final model, along with results of the CEA, are available in a separate publication (17). We compared long-term PRSS for people with substance use disorder (SUD) received after completing specialty SUD treatment (“the intervention”) to relying on the beneficial effects of specialty SUD treatment alone (“treatment as usual”). We proposed using quality-adjusted life years (QALYs) added by PRSS compared to treatment alone as one primary outcome of interest in order to ensure comparability of results with CEAs of other interventions, and we sought feedback from the RCCs on selecting a more salient primary outcome that would be more readily interpretable by future users of the calculator.

After developing a list of preliminary parameters gathered from the literature search and a preliminary set of formulas describing how each model component would be estimated, we converted each of these components into a plain language description of how each model component functions. For example, (Retp*Rp*Nt*D*Tt) was described in plain language as follows:

“Among people who get PRSS after specialty SUD treatment (*Nt*) and stay in it for at least a year (*Retp*), some percent of them will return to chaotic substance use (*Rp*), and of those who do return to chaotic use, we would expect a smaller percent to need to go back to specialty treatment (*D*) and incur that cost each time (*Tt*), so we want to capture that total cost. The percent of people who go to treatment who have SUD in any given year is drawn from the Substance Abuse and Mental Health Services Administration’s National Survey on Drug Use and Health for 2019 to avoid any unusual dips or spikes caused by COVID-19. If someone goes back to chaotic use, they would not be guaranteed to incur the costs of treatment – they’d just re-enter that same risk pool that everyone else with active SUD is in.”

The analysis for BND had already been conducted as part of a past study with which the research team was not involved (18). Coauthor MBM created a list of parameters from the original study publication (18) and updated each parameter to 2019 data. Throughout, 2019 was used as a focal date because of the impacts of the COVID-19 pandemic on substance use, overdose, healthcare, and general costs. MBM also worked to recreate the analytic model from the original study publication and converted each component to a plain language description as described for PRSS above.

**Figure 1:**
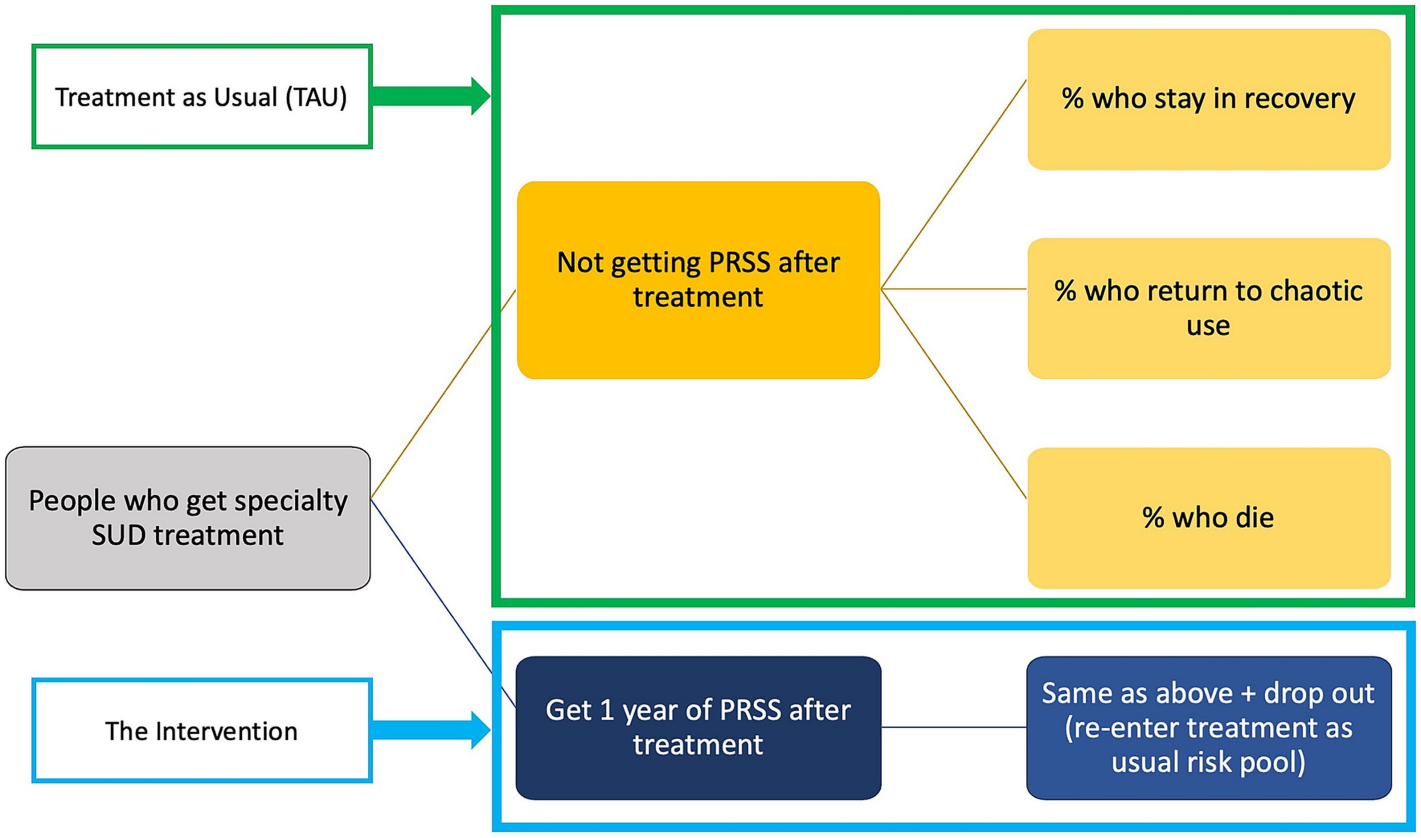
An example of a visual aid accompanying the plain language analytic model feedback session. This specific slide illustrates the microsimulation model, and how simulated participants would flow through the model, including the mutually exclusive and collectively exhaustive health states.

PowerPoint slides for a presentation to community partners were prepared for each model component for both models. These slides acted as visual aids to accompany plain language descriptions of each model component, an example of which is provided as Figure 1. In addition to soliciting feedback live during a feedback session (described in 2.3), we also prepared a brief anonymous online survey to capture written feedback if community partners preferred to share that way.

### 2.3 Analytic model refinement with community partners

The first of two feedback sessions focused on the analytic models and was scheduled for 90 minutes. Sessions were held separately for each RCC via Zoom and were recorded to ensure all feedback was captured.

#### 2.3.1 Economic evaluation 101

The first 30 min of the first feedback session consisted of a plain language primer on economic evaluation, with a particular focus on CEA. The goal was to equip community partners to engage with the model feedback process and to eventually prepare them to use and interpret calculator results. As an additional benefit, community partners may also be better able to interpret the results of other economic evaluations they encounter outside of using the calculator.

The economic evaluation primer began with an explanation of the kinds of questions these methods seek to answer. Methods like cost–benefit analysis and return on investment were briefly introduced, and then the remainder of the time was spent on CEA. We introduced the basic formula for CEA, the typical perspectives on cost (health system and societal), how we determine what outcomes to use as a denominator, and how to interpret incremental cost-effectiveness ratios (the primary result of a CEA). We introduced both standard or conventional approaches such as those detailed in the Second Panel on Cost-Effectiveness (23), as well as identifying areas where community input was essential to shape how the analysis would proceed, as is recommended in the recent updates to CHEERS (19). An example of this primer on economic evaluation is available on the calculator website^1^ as a recorded tutorial.

#### 2.3.2 Plain language model walk-through

As described above, we had converted each model component into a plain language description and provided a visual representation of model components in PowerPoint slides. After completing the primer, we walked through each parameter list and model component for both PRSS and BND. We solicited feedback after introducing each component, and after covering the parameters. We also solicited feedback on two key elements of the analysis: selection of a primary outcome of interest in addition to QALYs, and selection of a willingness-to-pay (WTP) threshold that was more salient to the intervention’s stakeholders than the standard $50,000, $100,000, and $200,000 per QALY WTP thresholds commonly in use (24, 25). Finally, we also asked about potential user inputs for the forthcoming prototype calculator, and whether those inputs would be reasonable for RCCs to input on their own.

In addition to recording the live session and taking notes, we also invited community partners to provide optional anonymous feedback via online survey provided at the end of the session and as a follow-up emailed link. Anonymous online surveys for the first session remained open until the second live session. We asked about any other health-related outcomes to use as a primary outcome of interest, in addition to QALYs, any other economic outcomes of interest (in addition to averted medical costs), anything missing from the model that would be important to capture, and any additional comments or questions. We also included a single multiple-choice item (Likert-type) to see if the community partner felt they better understood economic evaluation after the feedback session’s economic evaluation primer. Completing this anonymous online survey was optional for all community partners and there was no compensation.

### 2.4 Prototype calculator development

After incorporating feedback on the analytic models, SCM, MBM, and HSB developed prototype calculators in Excel. Separate prototypes were developed for PRSS and BND. Each prototype included an input and results sheet that would mimic what calculator users would see on a future web-based calculator. The remaining sheets—at least one for background cost calculations and at least one for background QALY calculations—would perform the analysis from the final analytic model based on numbers input into the corresponding cell on the input sheet. After completing the calculations, several key results would be displayed on the input and results sheet. For the prototypes, these results included: incremental costs, incremental effects in terms of QALYs added and in terms of the primary outcome selected by community partners, and incremental cost-effectiveness ratios (ICERs) for both QALYs and the primary outcome. Each ICER then had a set of plain language interpretation logic checks: a yes or no output indicating whether the ICER fell below each of the WTP thresholds, and—if the ICER was negative—whether the ICER was negative because of a negative numerator (the intervention was lower cost than treatment as usual) and positive denominator (the intervention produced more benefits than treatment as usual) which can be interpreted as meaning the intervention is both cost-saving and cost-effective (26).

**Figure 2:**
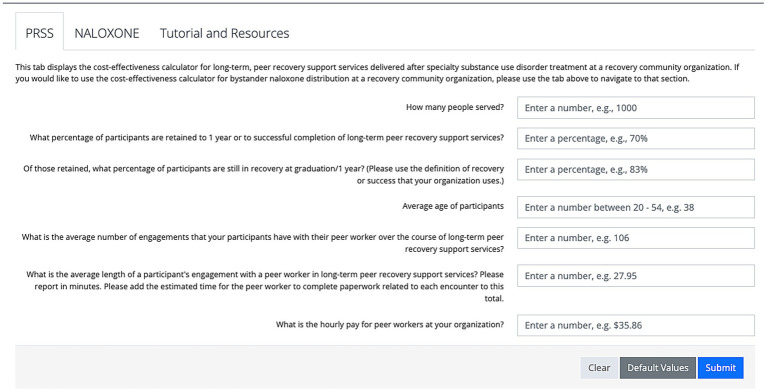
The calculator user interface prior to entering any user input information. The default component displayed is for peer recovery support services (PRSS).

**Figure 3:**
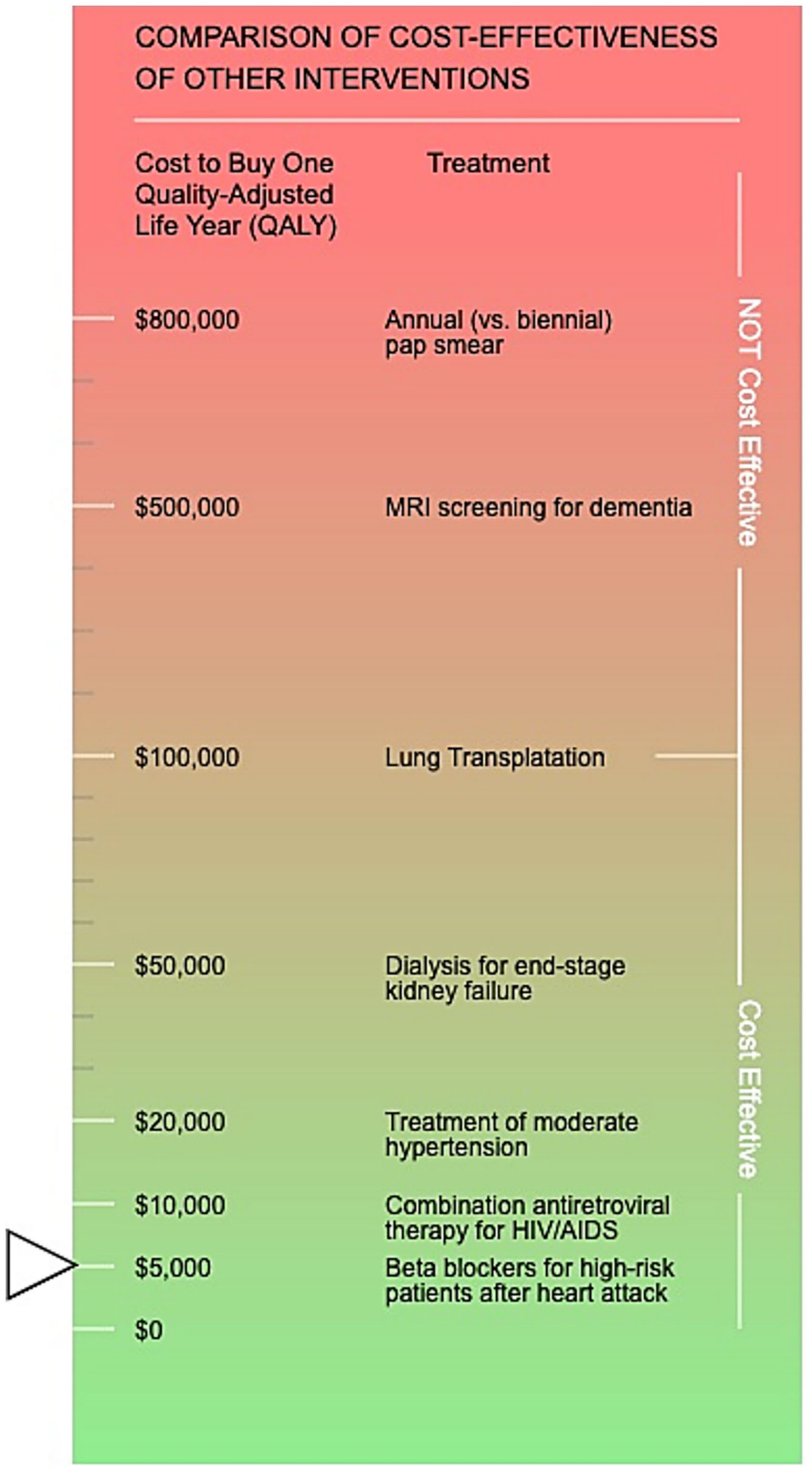
The visual aid displayed in the calculator results section demonstrating where your customized results incremental cost-effectiveness ratio in terms of quality-adjusted life years lands in comparison to other commonly-recognized health interventions. The societal perspective default values results are displayed (arrow).

After developing the prototype calculators in Excel, HW then converted the Excel spreadsheets to HTML code and built a web-based prototype of the calculator with all background calculations are hidden from the calculator user. The user only sees the user input fields and the descriptive text accompanying each field. There are three buttons below the input fields: “clear,” “default values” which inputs base case values from the original analysis (updated to 2019 values in the case of BND), and “submit.” The only input that does not truly default to the base case PRSS CEA (17) parameter is the number of people served in 1 year: in the full CEA, we used the full U.S. population with SUD who received specialty treatment, while in the calculator we set the default value to an arbitrary 1,000 people. Figure 2 provides an example of the user interface for PRSS prior to the user entering any information. After hitting the submit button, results display similar to those described for the Excel-based prototype, with the addition of a QALY ICER comparison visual aid (see Figure 3). The visual aid shows an array of commonly-recognized health interventions, such as dialysis, lung transplants, and antiretroviral therapy for HIV/AIDS. The interventions are arranged in order of largest (at the top, in the red colored portion of the gradient) to smallest (at the bottom, in the green portion of the gradient) ICER in terms of QALY. A small arrow indicates where the user-input QALY ICER falls in comparison to those other interventions on the visual gradient. The color of the gradient changes from red (highest) to yellow to green once below $50,000 per QALY. The location of the arrow changes when inputs change and the user hits submit with new values.

Finally, for PRSS there are two results tabs: one for the health system perspective and one for the societal perspective. However, we opted to use only one perspective for BND—the healthcare system perspective—since most of the costs are traditionally borne in that system in the absence of BND.

### 2.5 Prototype calculator pre-testing

In preparation for the second feedback session focusing on the web-based calculator prototype, the prototype was made available only directly via a URL, which only the research team and community partners would have during the feedback session. We also prepared a second anonymous feedback form to deploy during the second feedback session. We asked multiple choice (Likert-type) questions about how difficult or easy it was for the user to use the calculator, understand the calculator results, and find the information needed to input into the calculator, as well as questions about the usefulness and potential benefits of using the calculator for their organization. We also included several open-ended items requesting any additional comments about using the calculator, understanding the calculator, or anything else.

The second feedback session was also held via Zoom, with separate sessions for each organization, and included the same community partners as the previous feedback session but was scheduled for only 60 min. The session was recorded, notes were taken of feedback given live, and community partners could share written anonymous feedback via an online survey link that was shared at the end of the session, and via email as a follow-up after the session. We kept the anonymous feedback survey open for 1 month after the second session.

To gather feedback, we walked through each piece of the prototype web-based calculator, pausing to gather feedback on each component. We asked for feedback on the wording of helper text accompanying each user input field, as well as feedback on the wording of all results.

### 2.6 Pilot calculator launch

After the second session, all feedback gathered from community partners was integrated into the prototype web-based calculator. We then created an online survey to gather additional feedback from calculator users after launch. We asked about the ease of using the calculator, ease of interpreting results, ease of finding information to input into the calculator, and the usefulness of the calculator to the person’s organization. We refined the Economic Evaluation 101 primer portion of the first feedback session to be suitable as a recorded tutorial and for use in future dissemination and training efforts with other RCCs. After finishing the full CEA of PRSS and incorporating the results of that analysis into a presentation to accompany the tutorial (17), we then fully launched the pilot calculator at the National Association of Peer Supporters (NAPS) national conference in October of 2022. Because the NAPS conference was in person with no hybrid option and thus was not recorded, we presented similar material at a recorded webinar shortly afterward, and that recording became part of the final pilot calculator website as a recorded tutorial. The feedback form remains open and will continue to gather feedback on the final pilot calculator from calculator users until work on additional components and refinement of existing components begins in the future.

## 3 Results

### 3.1 Community partner engagement

Attendance at feedback sessions was consistent across both sessions and both organizations. Only one community partner member opted to use the anonymous feedback form 2 days after the first feedback session. Otherwise, all feedback was provided live during each session. In addition to the feedback gathered live during the first session, the anonymous respondent also indicated that self-reported recovery capital is an outcome of great interest across RCCs like theirs. They also indicated that they “somewhat agree” that they better understood economic evaluation after the primer.

#### 3.1.1 Calculator development

The calculator was launched in October 2022 and is available currently at https://go.uth.edu/cea. The primary outcome of interest selected for PRSS as an alternative to QALYs added was the number of people in recovery at 3 years after initiating long-term PRSS compared to specialty SUD treatment alone. Both this alternative primary outcome and the traditional CEA outcome of QALYs were estimated using a microsimulation approach, with ((1-*Rp*)*Retp*Nt) as the basic formula to estimate the number retained in recovery in the initial year post treatment. *Nt*, *Rp* and *Retp* were estimated using user input values from the calculator for the first year after specialty SUD treatment [but see the companion publication (17) for details on parameters used in the underlying analysis, which are also used as “default values” in the calculator, except for *Nt*, which uses an arbitrarily-set number as the default]. *Nt* was the number of people served in 1 year, *Retp* was the percent of those retained to 1 year or to successful completion of PRSS if before 1 year, and *Rp* was the percent of *Retp* who were still considered in recovery at 1 year or at completion if before 1 year. In the microsimulation to estimate outcomes (QALYs added and number of people retained in recovery at 3 years), *Rp* is used directly from user input values for the first year. For years 2 and 3, *Rp* reverts back to the stage transition probabilities for the probability of transitioning from the “recovery” health state to the “active SUD” health state that were used in the full CEA (17), which were drawn from a longitudinal study of changes in the probability of remaining in recovery with additional years of continuous recovery maintenance (27). That study found that after maintaining continuous recovery for 3 years, the probability of returning to chaotic substance use stabilizes long-term (27), thus for years after year 3, the probability of remaining in the “recovery” health state remains at the year 3 probability for the remainder of the simulation, except to accommodate growing mortality risk as the simulated participant ages. These mortality rates include normal background mortality by age for the U.S. population, and also incorporate the additional risk of mortality for people with SUD (28–31).

Importantly, in our original analysis, the recovery parameter (*Rp*) was operationalized as either abstinence from substances or as a sustained reduction in use in the original study from which we drew the parameter estimate (17, 32). The consequent reductions in healthcare utilization and societal costs like criminal legal system involvement are drawn from the same study, thus this broader operationalization of recovery as an outcome is correctly matched to one-year health system and societal cost savings. However, it is worth noting that calculator users were encouraged to use the definition of recovery from their own programs, which may include programs that use an abstinence-based definition of recovery, which could potentially lead to a mismatch in estimates of averted costs. Because an abstinence-based definition of recovery could be considered stricter and more restrictive than one that includes sustained, reduced use, this mismatch would likely lead to an overly-conservative, under-estimate of cost savings, thus is preferable to a scenario that over-estimates savings.

The average age of participants is a user input value that is not used in the full CEA [average age of U.S. SUD population (33, 34) is used], but only in the calculator. The calculator begins the microsimulation in the appropriate 5-year age bracket indicated by the user input. This allows for tailored mortality risk to be accurately applied based on the average age of participants at that specific RCC. This is key as some centers may specifically target a younger or older population depending on the surrounding community and other community resources. Currently, the calculator can accept average ages between 20 and 54 and will simulate changes in life expectancy as the result of the intervention or treatment as usual up through age 82. This feature is only present in the PRSS component, as we did not expect calculator users would know the average age of those who bystanders helped with their naloxone. After the simulated participants enter the microsimulation at the indicated age, the numbers of individuals who stay in recovery, return to chaotic substance use, or die are estimated each year using the stage transition probabilities previously described. This process repeats each year through age 82 (approximate life span) and total life years are tallied. Life years are then adjusted for quality of life, with SUD quality of life being estimated as an average of all disability weights from the Global Burden of Disease Study, which provides disability weights by SUD type (35). Because there was no estimate of the quality of life weight for the recovery health state available in the literature, we estimated that weight to be an average of general U.S. adult quality of life [0.86, (36)] and the least-impactful form of SUD from the Global Burden of Disease study (35): mild alcohol use disorder (0.741 when converted to a utility weight). Once quality of life adjustments are made and thus QALYs are estimated, 3% discounting per year is applied for all QALYs gained after year 1. Thus, the final estimate of QALYs added by the intervention are discounted for time, but the estimate of people retained in recovery at year 3 is not discounted (as it is not common practice to discount number of individuals).

The default values for *Rp* and *Retp* are drawn from an evaluation of long-term PRSS that operationalized recovery as either abstinence from substances *or* sustained reduced substance use, which our community partners advised was in line with the practices of many RCCs and the ethos of PRSS, which values participant-directed definitions of recovery and individualized recovery goals.

The PRSS portion of the calculator also has three user input values that will estimate a typical PRSS episode cost per participant: the average number of engagements that a participant has in 1 year (or to successful completion of their long-term PRSS engagement if less than 1 year), the average length of each session (including time for documentation), and the hourly pay for peer workers.

The PRSS results section of the calculator displays societal and health system perspective results for total and per-person averted societal or medical costs, respectively. It also displays incremental effects—or how much more benefit the intervention condition produced compared to treatment as usual—and breaks QALYs added into a per-person value, as well as providing total QALYs added. ICERS are interpreted in plain language using the logic checks described in section 2.4.

The BND calculator component has a much simpler user interface compared to the PRSS component, as it only uses the health system perspective, and because there are fewer inputs. The two user inputs on the BND component are the cost of naloxone, and the percentage of RCC participants to which the user wishes to distribute naloxone. Because an estimated 20% of bystanders likely to witness an overdose are already are in possession of naloxone (18), the calculator adds 20% to the user input, and can take a maximum value of 80% if the user wants to give naloxone to all RCC participants who do not already have naloxone. The primary outcome in addition to QALYs is the number of people whose lives are saved by the bystander naloxone compared to waiting for first responders to arrive.

### 3.2 Post-launch feedback

While the calculator had 269 visitors in just a four-month span between April and August 2023, the pace of feedback form completion is much slower by comparison, with only 24 forms completed since the October 2022 launch. Results of the quantitative items ranking ease of calculator use, ease of understanding results, ease of finding user input information, and usefulness and potential benefit of using the calculator for the user’s organization are presented in Table 1. Most found the calculator easy to use, easy to understand the results, but did have some difficulty finding information to input. Open-ended responses were also generally positive. Respondents indicated a desire to receive additional training for themselves and their colleagues to more effectively use the calculator. One respondent commented on the choice to frame the long-term PRSS CEA as delivered after specialty SUD treatment, rather than in comparison to no specialty SUD treatment, as is often the case.

**Table 1:** Results of the post-launch feedback form (*n* = 24).

Item	*n*	%
Please rate how difficult or easy you felt it was to use the calculator		
Very difficult	0	0
Somewhat difficult	1	4.17
Neither easy nor difficult	2	8.33
Somewhat easy	14	58.33
Very easy	7	29.17
Please rate how difficult or easy it was to understand the results that the calculator produced		
Very difficult	0	0
Somewhat difficult	2	8.33
Neither easy nor difficult	6	25.00
Somewhat easy	11	45.83
Very easy	5	20.83
Please rate how difficult or easy it is to find the information you need to enter into the calculator		
Very difficult	2	8.33
Somewhat difficult	1	4.17
Neither easy nor difficult	9	37.50
Somewhat easy	10	41.67
Very easy	2	8.33
Please rate how useful the results of the calculator are or how useful you think they may be to you or your organization		
Not very useful	0	0.00
Somewhat unuseful	0	0.00
Neutral	5	20.83
Somewhat useful	10	41.67
Very useful	9	37.50
Please rate your agreement with the following statement: I feel confident using the results of the calculator for the benefit of my work or my organization (for example, using the results in a grant proposal, in a report to stakeholders, to evaluate your programs, etc.)		
Strongly disagree	0	0.00
Somewhat disagree	2	8.33
Neither agree nor disagree	5	20.83
Somewhat agree	11	45.83
Strongly agree	5	20.83

Finally, the calculator and the accompanying tutorial presentation have continued to be presented to audiences across the US in more than a dozen webinars, at which the primary audience has been RCCs and similar organizations that deliver peer-driven substance use interventions.

## 4 Discussion

The slightly greater challenges in finding the information to input into the calculator (see Table 1) can be partly explained by challenges in data platforms and electronic health records software available to RCCs. Both community partners used data platforms that—at the time of co-creating the calculator—made it relatively easy to find the information needed for the user input fields. However, there are multiple available data platforms and electronic health records systems available for RCCs to use, and these platforms may also undergo updates and changes that render the user input information more challenging to find. Previous research on RCCs has found variability in RCC data collection and maintenance infrastructure and philosophy (20), thus this challenge can be expected to persist among potential calculator users in future iterations.

Community partners echoed the one anonymous survey respondent’s questions about the comparison of PRSS plus specialty SUD treatment to specialty SUD treatment alone. They noted that, in some cases, long-term PRSS may allow participants to bypass other types of treatment entirely or may lead people to engage in more formal types of treatment. In discussions with the community partners, the potential pathways from treatment, to treatment, or around treatment that PRSS presents are unfortunately insufficiently understood to model currently. One previous study found that about 17% of referrals to RCCs came from treatment (20), but other pathways (e.g., initiating care via PRSS at an RCC, then later going to treatment, or bypassing treatment) are not yet well understood, and should be the subject of future research.

Research on PRSS and RCCs continues to advance, including the recent publication of a cost analysis of two types of PRSS provision within a single organization (37). Such research is critical to not only establishing key parameters for more accurate economic evaluations of variable PRSS models within heterogeneous settings, but may also help address known challenges with consistent data collection described above. For example, in the PRSS model run by paid peer workers in the recent cost analysis, administrative labor costs—the category that includes but is not limited to data collection and management—comprised about 20% of the total program costs, while in the volunteer-driven PRSS program, these costs were 46% of the total costs (37). Future research should assess RCC and other PRSS provider data collection and data management needs, with special attention to alignment with the philosophy and unique practices of PRSS provision.

### 4.1 Limitations

This was a relatively small pilot project that involved only 10 staff and administrators at 2 RCCs in the same geographic area, thus potentially limiting generalizability. While the calculator’s reach has expanded substantially since that time due to it being web-based, and due to the dozens of additional presentations since its launch, passive feedback-gathering has resulted in minimal responses (*n* = 24), though these responses are key to informing future efforts to improve and expand the calculator. Still, despite these limitations, this project demonstrates that community-based organizations engaged in delivering peer-driven substance use interventions can and have meaningfully engaged in economic evaluations and can substantively inform economic evaluation research and the development of tailored economic evaluation tools.

### 4.2 Conclusion

Substantial gaps remain in the economic evaluation literature for peer-driven substance use interventions. As these interventions continue to expand nationwide to address the ongoing overdose public health emergency, it is essential that those impacted by substance use and on the front lines of addressing its consequences are meaningfully involved in research that impacts them and their work. While this ethos of “nothing about us without us” must carry through to all research approaches, economic evaluation research has not traditionally embraced this approach. This project demonstrates that CBPR approaches to economic evaluation research are feasible—even with extremely limited funding—and can not only produce high-quality academic work (17) but can also develop tools to make economic evaluation information more accessible.

## Data Availability

The raw data supporting the conclusions of this article will be made available by the authors without undue reservation.

## Footnotes

^1^ https://go.uth.edu/cea
